# Metal artefact reduction of different alloys with dual energy computed tomography (DECT)

**DOI:** 10.1038/s41598-021-81600-1

**Published:** 2021-01-26

**Authors:** Anand John Vellarackal, Achim Hermann Kaim

**Affiliations:** 1 University of Basel, Petersgraben 35, 4001 Postfach, Basel, Switzerland; 2 Institute of Radiology, Hirslanden Klinik Birshof, Reinacherstrasse 28, 4142 Münchenstein, Switzerland

**Keywords:** Musculoskeletal system, Medical research

## Abstract

To evaluate the influence of dual-energy CT (DECT) and Virtual monochromatic spectral (VMS) imaging on: (1) the artefact size of geometrically identical orthopaedic implants consisting of three different compositions and (2) the image quality of the surrounding bone, three similar phantoms—each featuring one femoral stem composed of either titanium, chrome-cobalt or stainless steel surrounded by five calcium pellets (200 mg hydroxyapatite/calcium carbonate) to simulate bony tissue and one reference pellet located away from the femoral stem—were built. DECT with two sequential scans (80 kVp and 140 kVp; scan-to-scan technique) was performed, and VMS images were calculated between 40 and 190 keV. The artefact sizes were measured volumetrically by semiautomatic selection of regions of interest (ROIs), considering the VMS energies and the polychromatic spectres. Moreover, density and image noise within the pellets were measured. All three phantoms exhibit artefact size reduction as energy increases from 40 to 190 keV. Titanium exhibited a stronger reduction than chrome-cobalt and stainless steel. The artefacts were dependent on the diameter of the stem. Image quality increases with higher energies on VMS with a better depiction of surrounding structures. Monoenergetic energies 70 keV and 140 keV demonstrate superior image quality to those produced by spectral energies 80 kVp and 140 kVp.

## Introduction

Postoperative imaging of arthroplasty is crucial to identify complications early on. One of the most frequently used and powerful postoperative imaging modalities is Computed tomography (CT)^1^.

However, CT imaging of arthroplasty implants may produce strong artefacts such as beam hardening, partial volume and aliasing, scatter and streak artefacts that reduce the quality of the image^2,3^. There are two main reasons which typically explain the occurrence of metal artefacts: beam hardening and photon starvation^4^.

There are different parameters that can be changed to overcome artefacts in conventional multidetector CT imaging including rise of the tube current^5,6^, increase of the tube kilovolt peak and using thin collimations and a low pitch. During image reconstruction thick sections, smooth reconstruction filters (kernel), and the use of an extended CT scale of Hounsfield units (HU), if available, will diminish the degree of metal artefacts^6,7^.

In addition to conventional iterative image reconstruction (IR) algorithms, special and dedicated metal artefact reduction (MAR) algorithms have been introduced to apply interpolative methods to corrected IR data and raw data (spectral energy data set)^1,4,5^.

A very effective method to reduce beam hardening metal artefacts is the Virtual monochromatic spectral (VMS) imaging with dual-energy CT (DECT)^1,6,8^. DECT uses two datasets of the same anatomical area recorded at various X-ray peak kilovoltages (e.g. 80 kVp and 140 kVp)^9–11^. Because varying material composition alters energy-dependent attenuation of X-rays beams, the two datasets will display different attenuations profiles. The VMS image can be reconstructed and calculated with different photon energy levels between the spectrum from 40 to 190 keV with the help of monoenergetic extrapolation^12,13^. The VMS images have the potential to reduce beam hardening artefacts and ascertain the narrow energy range of radiation produced by the X-ray source—measured in kiloelectron volts (keV)^6^.

The aim of this experimental in-vitro study was to evaluate the influence of DECT and VMS imaging on: (1) the artefact size of geometrically identical orthopaedic implants consisting of three different compositions (titanium, chrome-cobalt and stainless steel) and (2) the image quality by considering the density of bone-equivalent and calibrated calcium pellets positioned around the implants. The data were acquired with self-constructed phantoms and sequential, scan-to-scan DECT.

## Materials and methods

### Phantom design

This experimental phantom study did not require prior approval by an ethics committee. A total of three similar phantoms were built, differing only in the composition of the femoral stem.

**Figure 1:**
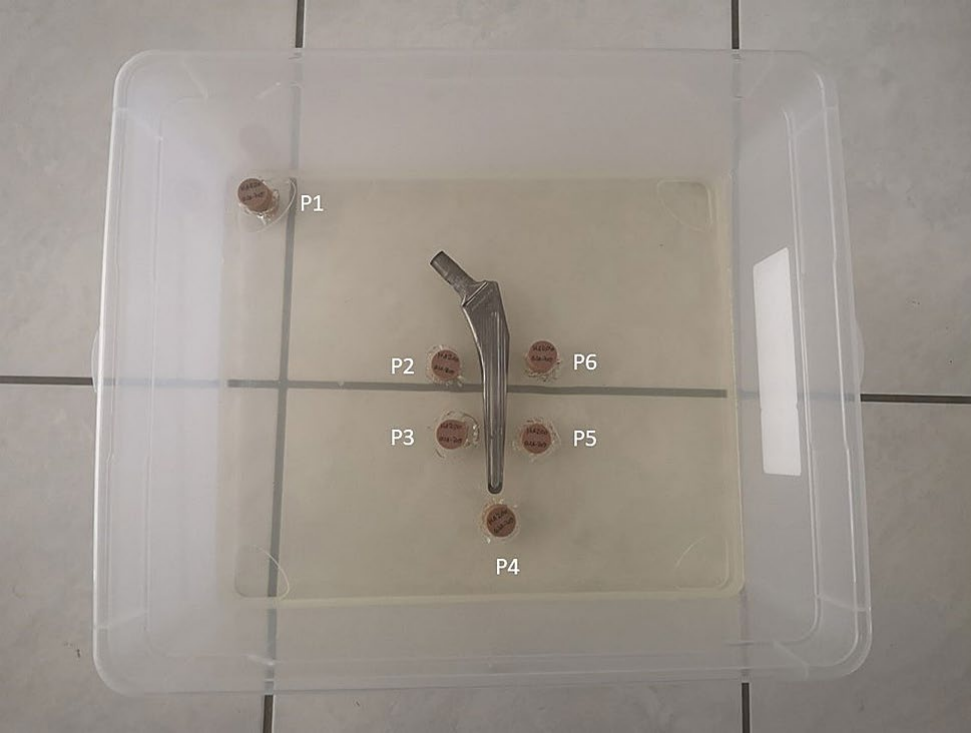
Image illustrates a phantom with implanted metal alloys. The construction was made for stainless steel, chrome-cobalt and titanium. Six calcium hydroxyapatite pellets (P1–P6) were implanted. Five pellets are surrounding the femoral stem and one pellet was placed far away from the implant and was used as the reference value.

The femoral implant of a hip prosthesis was placed within the centre of a rectangular plastic box with a dimension of 40 × 33.5 × 17 cm. Distances to the box walls were defined and reproducible. The implants were circumnavigated by five cylindrical, calibrated calcium hydroxyapatite pellets (200 mg of hydroxyapatite/calcium carbonate; HA/CC, from QRM, Möhrendorf, Germany) placed in a predefined positions around the prosthesis according to Gruen et al. 1979^14^: Zone 2, Zone 3, Zone 4, Zone 5, Zone 6, with a distance of 5 mm to the surface of the stem. A sixth pellet was positioned away from the implant at a box corner to avoid interference and serve as a reference value (Fig. 1) Each pellet was 30 mm in height and 20 mm in diameter.

To achieve stable suspension and position of the devices the box was filled with gelatinous water (5%).

The femoral stems consisted mainly of titanium, cobalt chrome and stainless. All implants (Zimmer Biomet, Winterthur, Switzerland)^15^ were of identical shape, size, surface character and geometry:

1. Protasul S30 is very hard iron-based forging alloy with high content of chrome and nickel. (*Stainless steel implant*)
2. Protasul 100 is a heavy-duty forging alloy composed of titanium, aluminium and niobium. (*Titanium implant*)
3. Protasul 10 is heavy-duty forging alloy composed of cobalt, chromium, nickel, molybdenum and minor constituents of other substances. (*Chrome-Cobalt implant*)

### CT data acquisition

All examinations were performed on a single source CT scanner (Somatom Sensation 64, Siemens Healthcare Forchheim, Germany). To achieve Dual Energy CT (DECT) images, a scan-to-scan technique was used. Two sequential scans with 80 kVp and 140 kVp were acquired with adapted acquisition parameters to assure similar noise levels of both scans. The tube current–time products were 265 mAs (80 kVp) and 91 mAs (140 kVp), the exposure time 15.17 s, scan lengths 341 mm, pitch 0.6 mm, slice acquisition 64 × 0.6 mm. CT doses (DLP) were calculated to 24.87 mGycm (80 kVp) and 49.18 mGycm (140 kVp). There was no necessity for hard filtration. Acquisition FOV was 500 mm for both scans.

Standard iterative reconstruction (IR) (level 3, range 1–5) and a bone (sharp) kernel (Q70) were used for image reconstruction, with a slice thickness of 0.75 mm and an increment of 0.5 (image matrix 512 × 512). Postprocessing was performed with a monoenergetic application algorithm, which was proposed and installed on the scanner for routine use by the manufacturer (Siemens). Virtual monochromatic spectral (VMS) images were calculated at 40, 50, 70, 100, 120, 140 and 190 keV. The intervals between the 40 and 190 keV had been defined by the software and could not be arbitrarily varied. The two other datasets generated consisted of the originally acquired, polychromatic 80 kVp and 140 kVp images.

### Quantitative data analysis

#### Metal artefact segmentation

All data sets were evaluated by image postprocessing software (Fa. TeraRecon GmbH, Frankfurt, Germany). The volume artefact of the metal implant was segmented to adjusted HU-based intervals and volumetrically calculated with this software.

**Figure 2:**
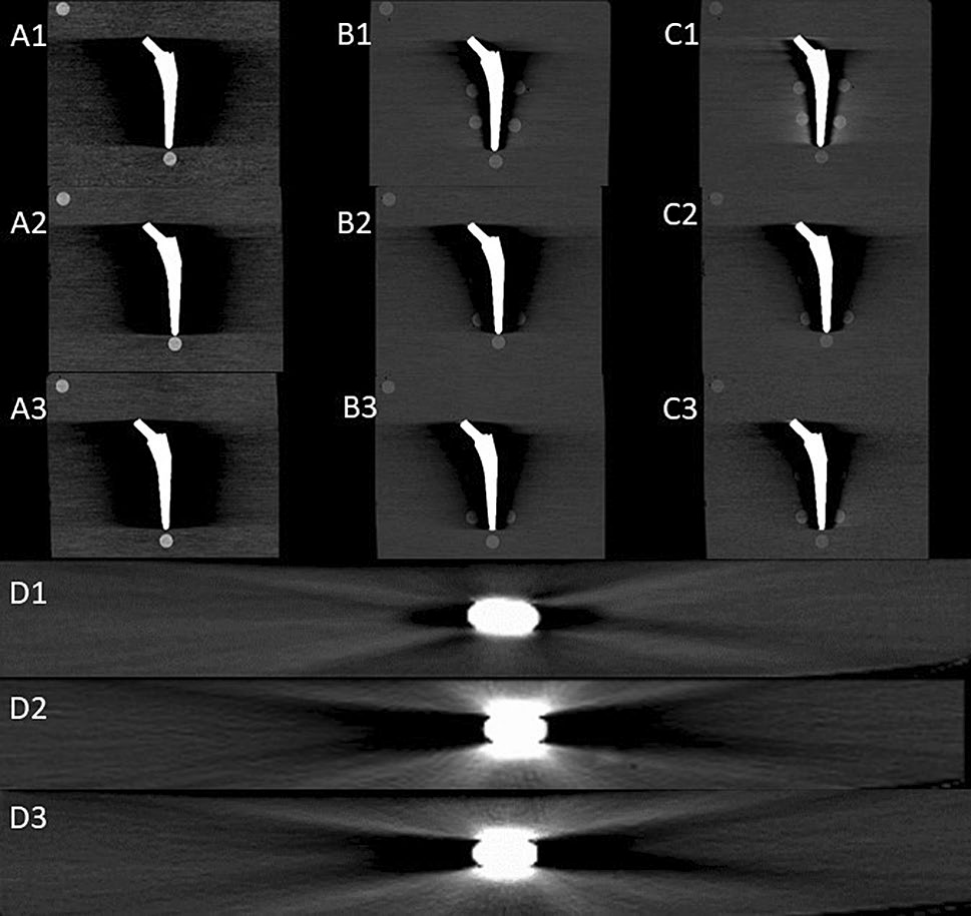
CT images of metal implants show metal artefact reduction with increasing energies 40 keV (**A**), 100 keV (**B**) and 190 keV (**C**). The grade of artefact reduction is similar with chrome-cobalt (2) and stainless steel (3) but increased with titanium (1). The transversal reconstructions (190 keV) on mid-stem level illustrate alloy-dependent dark and bright streak artefacts (**D**). The density of reference pellet 1 (left upper corner) is decreasing with rising energy.

The range of HU densities for dark and bright artefacts were defined by appreciating values for background noise on images with an expected maximum of heterogeneity (40 keV, 80 kVp), which were measured to −200 to 310, mean 17 HU respectively –200 to 330, mean 28 HU. Therefore, the values outside these ranges were attributed to bright and dark streak artefacts. (Fig. 2D).

The artefact size was measured volumetrically by semiautomatic region of interest (ROI) selection. The range of density was chosen between –1042 HU to –200 HU for the dark artefacts and 400 HU to 2500 HU for the bright artefacts. The volume of the prosthesis was subtracted from the value of bright artefacts to isolate the volume artefact value.

#### Linear attenuation coefficient (LAC)

**Figure 3:**
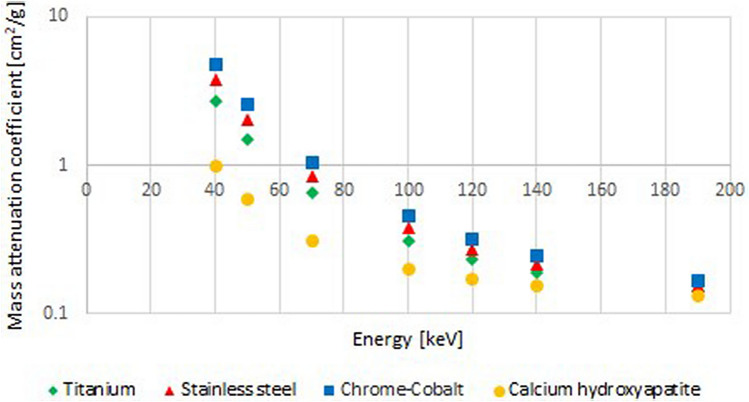
Graph illustrates the linear attenuation coefficient (LAC) in cmy^2^/g of titanium, chrome-cobalt, stainless steel and calcium-hydroxyapatite for different monoenergetic energies in keV.

The energy-dependent LACs (Fig. 3) of the three metallic alloys were calculated according to the specifications of the manufacturer^15^ by using weight fractions of the various components^16^.

#### Density measurement

The density of the attenuation in Hounsfield units and the image noise in standard deviation caused by the metal artefact were extracted utilizing software from Synedra (Synedra Information Technologies, Innsbruck, Austria) for monoenergetic and spectral energies. ROIs were defined as a circle (diameter 16 mm) and were placed over the centre of the 20 mm pellets, slice thickness 1 mm. The measurements with the specific ROIs were performed in all datasets in the same field size and on the same spot for all 6 pellets. The mean value and standard deviations were sampled for all acquired and reconstructed data sets considering all three phantoms.

#### Signal-to-noise ratio (SNR)

To ensure similarity and comparability, the signal-to-noise ratio (SNR) was calculated for all three different phantoms and reconstructed images. Hounsfield units of the sixth pellet was measured and divided by the standard deviation.

### Statistical analysis

All statistical analyses were conducted using python (Version 3.7, www.python.org).

Student’s t-test were used for the statistic plots with the p-value 0.05 for the error bar. To demonstrate the significant (p < 0.01) difference between monoenergetic and spectral energies a paired sample t-test were made for density deviation.

## Results

### Artefacts

The behaviour of dark and bright artefacts follows the same trend, independent of the identity of the metallic alloys or experimental energy level. The dimension of bright artefacts was about 10–20% of dark artefacts. The latter provoke the main interference of neighbouring structures which have been simulated by calibrated calcium hydroxyapatite pellets.

**Figure 4:**
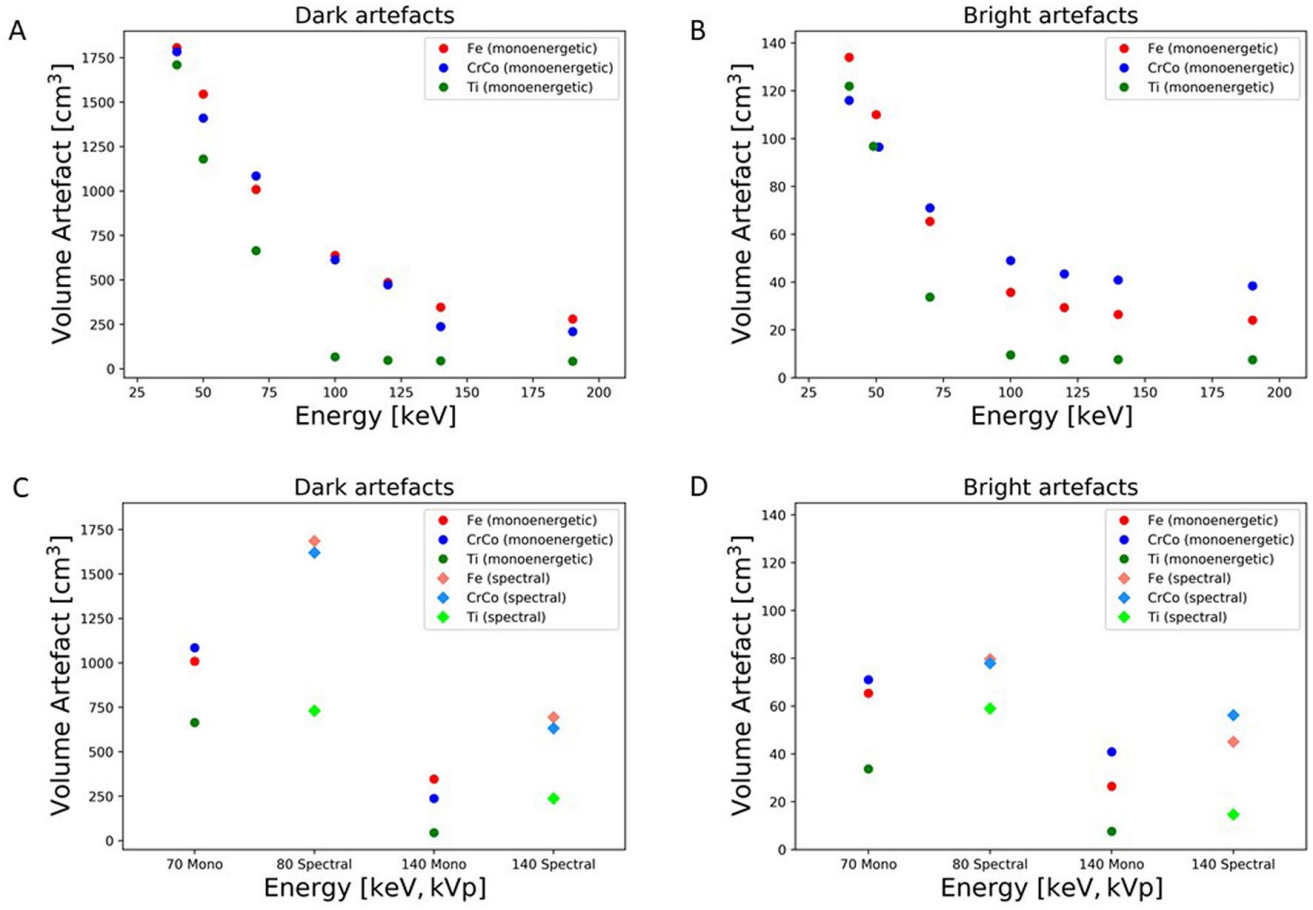
The graph illustrates the reduction of volume artefact with increasing energies keV for dark artefact (**A**) and bright artefact (**B**). The other two graph show the impact of volume artefact reduction between monoenergetic and spectral energies for dark artefact (**C**) and for bright artefact (**D**).

With all three metallic alloys the volume of artefacts was greatest with lowest energy (40 keV) and diminishes with rising energies (Fig. 2A–C). The graph of the energy-dependent artefact reduction is hyperbolic with significant alloy-dependent differences in the slope (Fig. 4A,B).

The titanium-induced beam hardening artefacts were downsized to a fraction of 4% for dark artefact and 8% for bright artefact with 100 keV (Dark artefact: 67 cm^3^, Bright artefact: 9.51 cm^3^), and the artefact volume is consistent between 100 and 190 keV. The reduction of dark artefact volume from 40 to 190 keV is 97.5%. Similarly, bright artefact volume reduction is 94% for the same energy range.

The decline of artefact volume with stainless steel and chrome-cobalt alloys runs parallel, with stepwise reduction until 190 keV. The artefact volume at 190 keV comprises about 15% (dark artefact) and 18% (bright artefact) for stainless steel and about 12% (dark artefact) and 33% (bright artefact) for chrome-cobalt of volume with 40 keV. The steps of volume reduction get smaller between higher energies (100 keV to 190 keV). (Fig. 4A,B).

The residual artefact size (the sum of dark and bright artefact) of stainless steel (304.1 cm^3^) and chrome-cobalt (248.4 cm^3^) implant is 6 or 5 times greater than that of titanium (50.5 cm^3^) at 190 keV.

The comparison of VMS 70 keV with spectral 80 kVp and VMS 140 keV with spectral 140 kVp documents the superior effect and artefact volume reduction with VMS (Fig. 4C,D).

### Pellets

Irrespective of the composition of the metal, dark streak artefacts extensively expand along and around the long axis of the metallic implant. Therefore, mainly the pellets 2, 3, 5 and 6 were either more or less strongly affected by the artefacts, whereas pellet 4 at the inferior and vertical end of the prosthesis was hardly affected. All density measurements within pellet 4 were similar to pellet 1 which served as the standard of reference. Due to implant geometry, pellets 2 and 6 presented greater effects from dark artefacts than pellets 3 and 5. (Fig. 2A–C).

### Density

**Figure 5:**
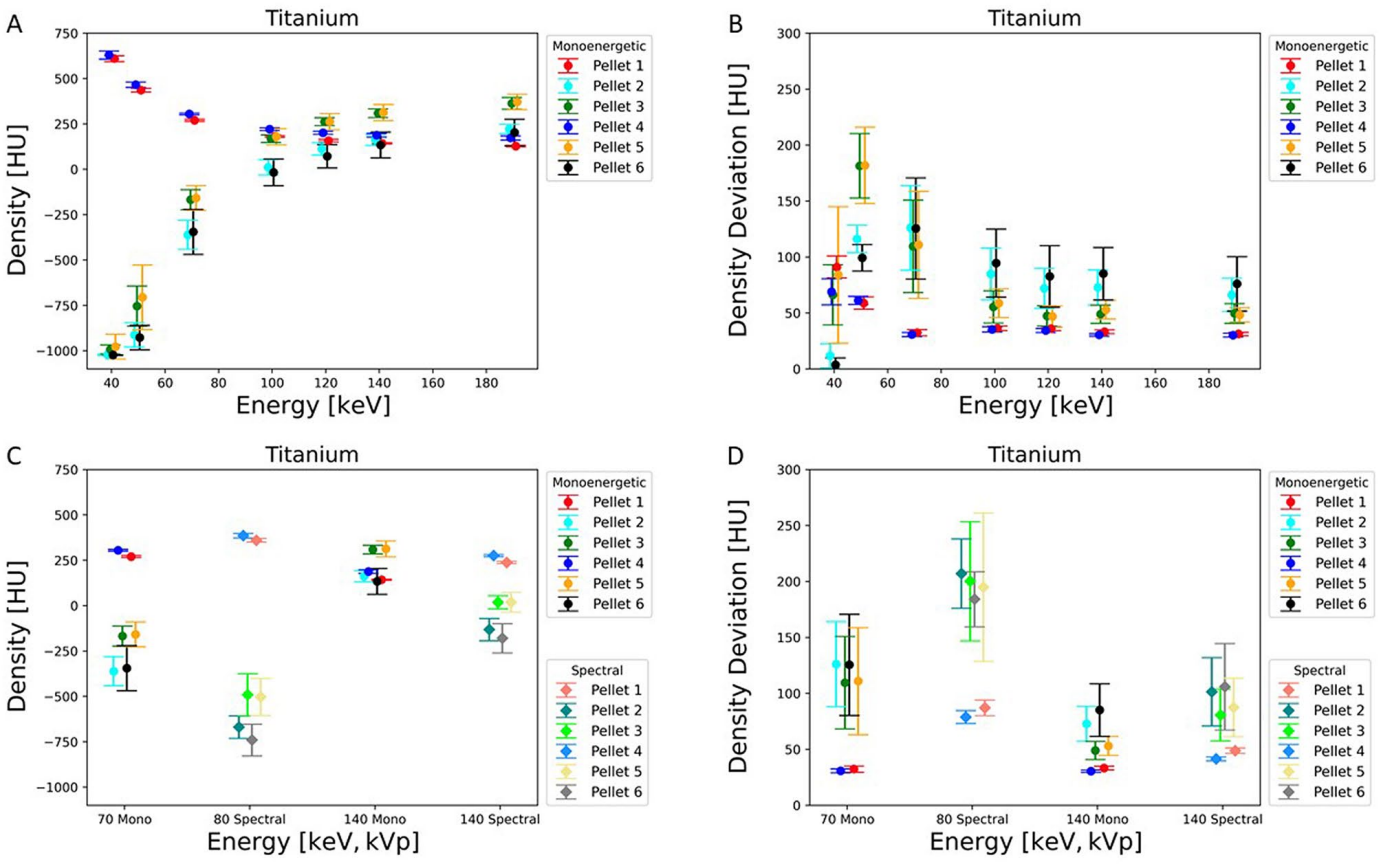
Graph demonstrates the density in HU (**A**) and density deviation in HU (**B**) of artefacts of the pellets close to the metal implant of titanium for different monoenergetic energies in keV. The other images show the density in HU (**C**) and density deviation in HU (**D**) of the difference between monoenergetic (70 keV and 140 keV) and spectral energies (80 kVp and 140 kVp) for titanium.

**Figure 6:**
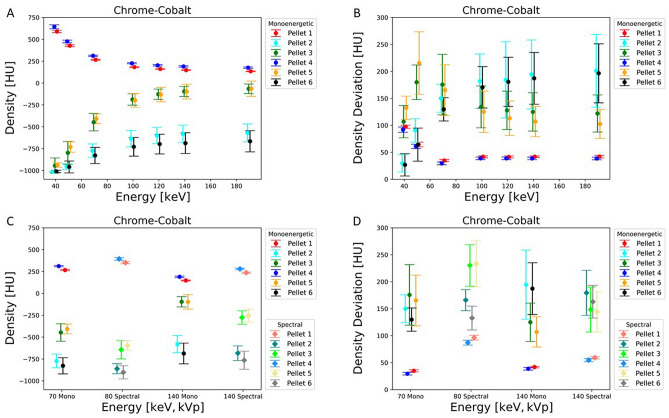
Graph illustrates the density in HU (**A**) and density deviation in HU (**B**) of artefacts of the pellets close to the metal implant of chrome-cobalt for different monoenergetic energies in keV. The other images show the density in HU (**C**) and density deviation in HU (**D**) of the difference between monoenergetic (70 keV and 140 keV) and spectral energies (80 kVp and 140 kVp) for chrome-cobalt.

**Figure 7:**
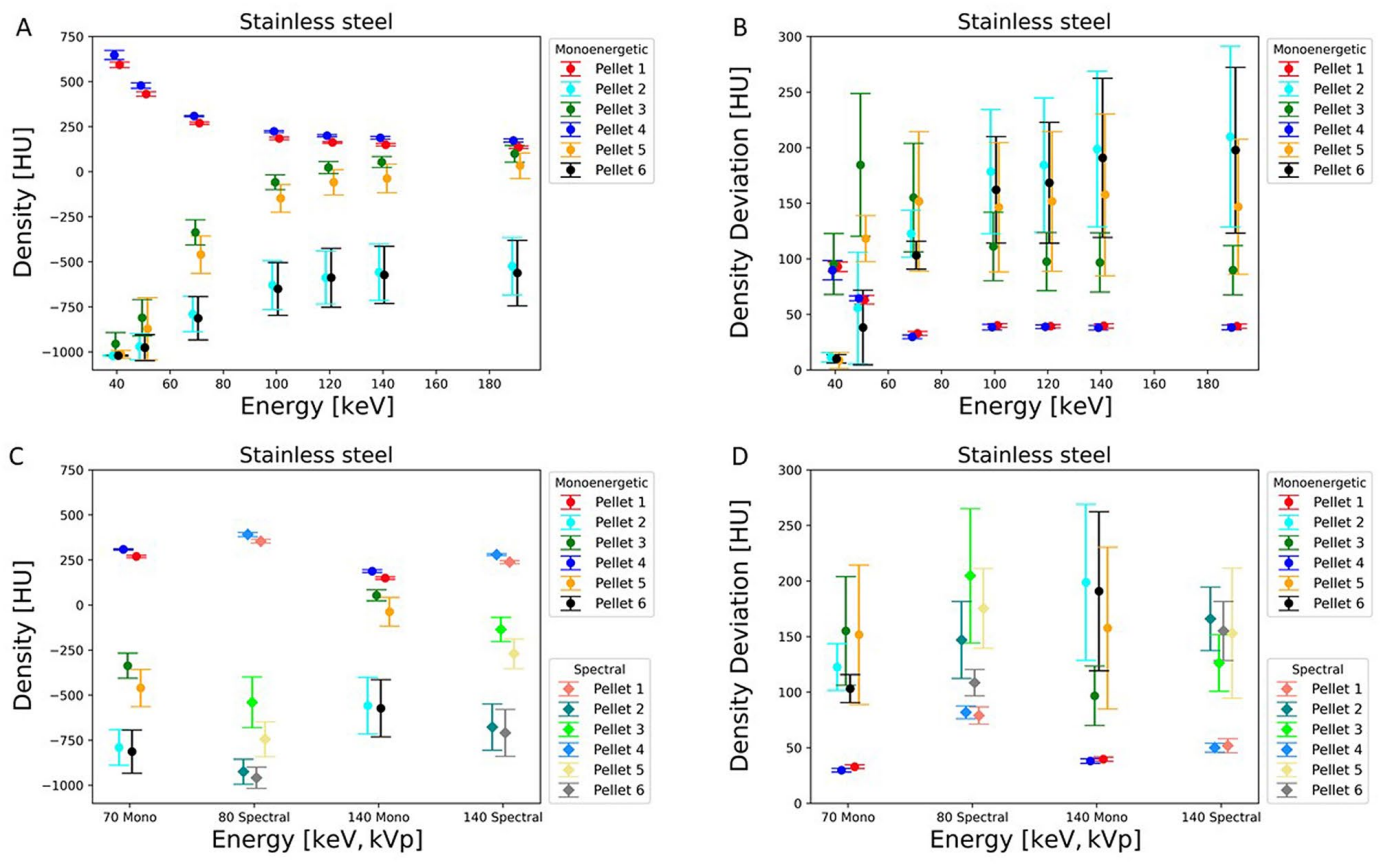
Graph demonstrates the density in HU (**A**) and density deviation in HU (**B**) of artefacts of the pellets close to the metal implant of stainless steel for different monoenergetic energies in keV. The other images show the density in HU (**C**) and density deviation in HU (**D**) of the difference between monoenergetic (70 keV and 140 keV) and spectral energies (80 kVp and 140 kVp) for stainless steel.

Density plots demonstrate for each pellet the mean value and its distribution in HU over eight consecutive slices dependent on variation of VMS energy. Basically, the lower the HU the more exists an overlay of dark artefacts affecting the ROIs, and the higher the HU the lesser dark artefacts affect the ROI over the pellets. Differences in density concerning one pellet in various measurements were dependent on energy level, monoenergetic or spectral energies and metallic alloy in the vicinity. (Figs. 5, 6,7A,C).

With all three metallic alloys strong and extended artefacts occur with 40 keV and 50 keV leading to densities between –700 HU to –1000 HU. Between 70 and 120 keV a significant rise of density (more than 400 HU) occurs, which concerns pellets 2, 3, 5, 6 with titanium prosthesis and mainly pellets 3 and 5 with chrome-cobalt (rise of 300 HU) and Fe alloys (increase of 350–400 HU).

With titanium all six pellets present with similar densities between 120 and 190 keV (100–250 HU) and even pellets close to the implant could be assessed without disturbance.

With chrome-cobalt and Fe alloys densities of pellets 3 and 5 approach the densities of pellets 1 and 4 between 120 and 190 keV (chrome-cobalt: –150 to 200 HU; stainless steel: –100 to 200 HU) without an impeccable, completely untroubled homogeneity, but influence of artefacts was significantly reduced. The effect seems to be even stronger with Fe prosthesis since the HU differences of pellets 3 and 5 to pellets 1 and 4 are less than with chrome-cobalt. Density levels of pellets 2 and 6, however, remain with strongly negative HU (chrome-cobalt: –650 to –550 HU; stainless steel: –600 to –500 HU) due to a persistent and substantial overlay of heavy artefacts.

An additional density plot illustrates values and differences from monoenergetic and spectral energies (70 keV vs 80 kVp and 140 keV vs 140 kVp). Beam hardening artefacts with strong influence on pellet densities are stronger with 80 kVp than 70 keV on all three experiments (Figs. 5, 6, 7C). With titanium, the distribution of densities is more homogenous with 140 keV (130–310 HU) than 140 kVp (–180 to 275 HU) due to substantial artefact reduction. It is noteworthy that density with spectral 80 kVp (350–400 HU) is higher than VMS 70 keV (270–350 HU) concerning pellets 1 and 4 for all three metal implants.

Concerning stainless steel and chrome-cobalt alloys, the effect of artefact reduction is stronger with monoenergetic 140 keV than spectral 140 kVp, evidenced by the density distribution of pellets 3 and 5 (chrome-cobalt reduction between 160 and 180 HU; stainless steel reduction between 190 and 220 HU). Density of pellets 2 and 6 are almost completely compromised by dark artefacts (–600 to –800 HU).

As basically expected, the density of reference pellet 1 gradually decreases between 40 and 100 keV due to the higher transmission of photons with increasing energies. The grade of X-ray absorption between 100 and 190 keV remains stable. (Figs. 5, 6, 7A).

### Density deviation

The interpretation of the measurements of density deviation is complex and defines the degree of artefact disturbance on the real density of each pellet. Results of density deviation were interpreted in conjunction with density plots (Figs. 5, 6, 7B, D).

The density deviation plots illustrate the mean value with standard variation of each ROI and each pellet, measured over eight contiguous slices. Therefore, the displayed values document not only heterogeneity within one ROI of a single slice, but also through plane heterogeneity over eight contiguous slices. A low-density deviation interval with low and negative HU means strong artefacts without pixels of original density within the pellet. A low-density deviation interval with positive HU close to the reference measurement of pellet 1 indicates a low grade of interfering artefacts. A high-density deviation with a broad standard deviation interval means a heterogenous distribution of density within the ROI and through the slices due to a mixture of areas superimposed or less disturbed by strong artefacts.

Irrespective of the metallic alloy, density deviations of pellets (2,3,5,6) were very low at 40 keV and 50 keV with density values (chrome-cobalt: –1020 to –750 HU; stainless steel: –1020 to –800 HU; titanium: –1020 to –700 HU) far in the negative range, indicating that all ROIs are intensely overlaid with dark artefacts (Figs. 5, 6, 7B).

With titanium alloy, the maximum of density deviation occurs with 70 keV, declining to 120 keV, and remaining stable to 190 keV. This is in conjunction with the density characteristics and due to an energy-dependent and successful artefact reduction (Fig. 5B).

A similar—but much less-pronounced—effect can be observed with pellet 3 and 5 close to the stainless steel and chrome-cobalt implants, whereas the density deviations of pellet 2 and 6 remain very high, indicating a heterogenous pixel and through-plane distribution due to dark artefacts. The density deviation patterns of all pellets in chrome-cobalt and stainless-steel experiments were very similar (Figs. 6, 7B).

The density deviations of pellets 1 and 4 show a moderate decline at energies between 40 and 70 keV due to a decrease of background noise with rising energies. The effect is a more homogenous density distribution. The distribution remains constant with energies between 70 and 190 keV. A similar phenomenon can be observed in the comparison of monoenergetic versus spectral energies (Figs. 5, 6, 7D) The latter consist of more heterogenous energy distribution at both levels (80 keV, 140 keV) resulting in an increased background noise level.

The difference in density deviation between monoenergetic and spectral energies was measured using the paired sample t-test. For all three metal implants the results were statistically significant between 70 keV and 80 kVp (p < 0.01). Between 140 keV and 140 kVp the difference in the density deviation was statistically significant for titanium (p < 0.01) while stainless steel (p = 0.77) and chrome-cobalt (p = 0.39) did not show a significant difference.

### SNR (signal-to-noise) ratio

**Figure 8:**
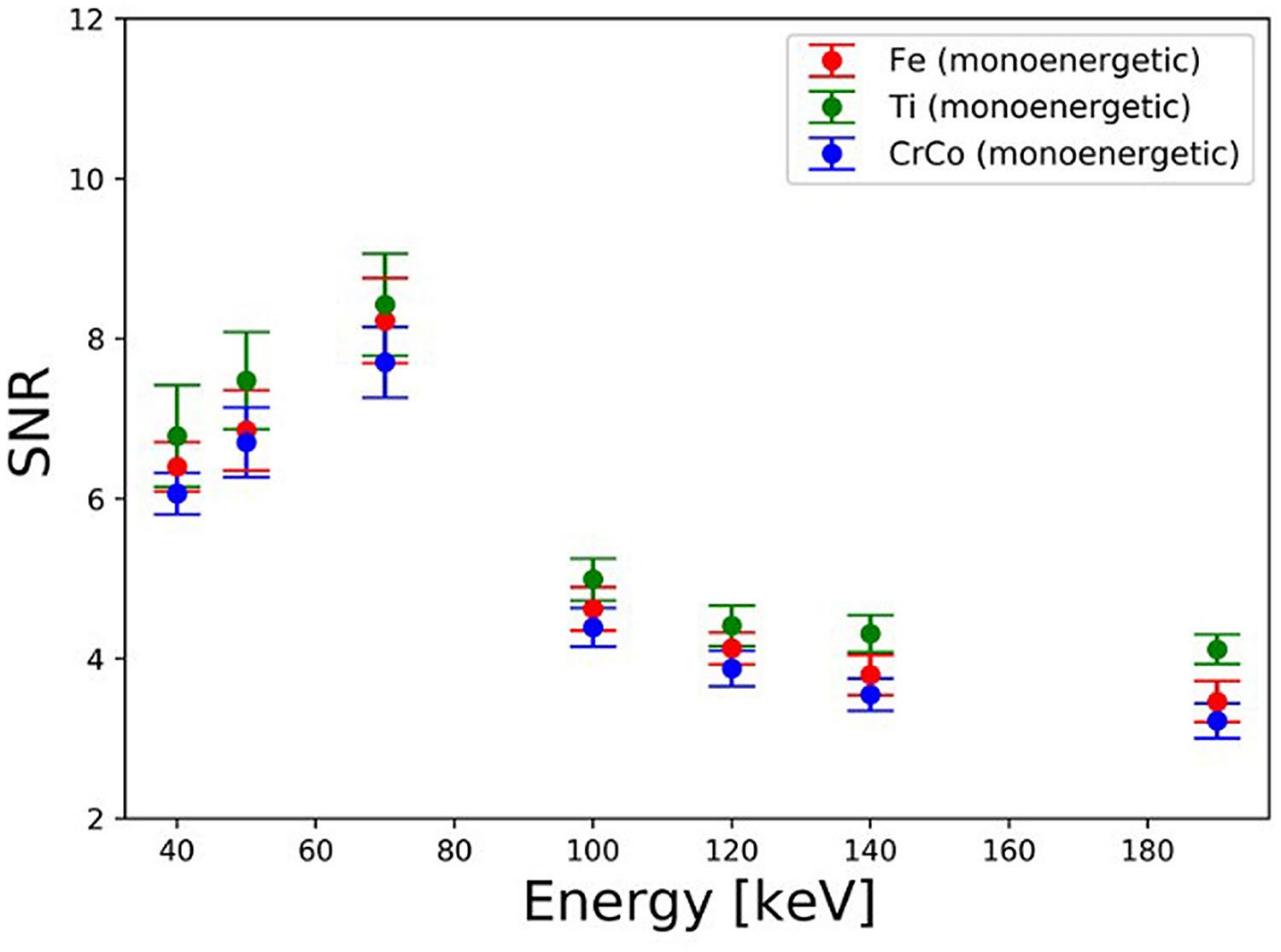
Image show the signal-to-noise ratio of three metal implants by different monoenergetic energies in keV.

SNR measurements of reference pellet 1 in the three experiments with differently compounded implants were similar at each energy level. The equivalence of SNR values assures comparability of all three examinations and value measurements. The distribution of values is as expected with a peak on 70 keV due to the optimal balance of background noise and photon absorption. The standard deviation for lower monoenergetic energies (40, 50 keV) is high because of the low quality and high background heterogeneity, whereas the decrease of photon absorption leads to a reduction of SNR with higher energies (100–190 keV) (Fig. 8).

## Discussion

### Metal artefact

This study investigated the effect of DECT with VMS on metal artefact reduction considering three different metallic alloys (titanium, stainless steel, chrome-cobalt), which are most commonly used in orthopaedic surgery.

There are several physical, helical, multichannel and patient-related causes for metal artefacts. The metal itself causes beam hardening, photon starvation, scatter artefacts and noise and the metal edges create artefact due to cone beam, undersampling, windmill and motion^2,3^.

In this study two evident and visually detectable kinds of artefacts (dark and bright artefacts, Fig. 2D) have been considered which are mainly created by the metallic composition due to beam hardening and photon starvation as the two major mechanisms. At energy levels used for diagnostic imaging, the X-rays get mainly attenuated by the photoelectric effect and Compton scattering. The grade of attenuation is influenced by the photon energy of the X-ray beam, the composition and thickness of the metallic hardware and can be characterized by the linear attenuation coefficient (LAC). The photoelectric effect is proportional to (*Z*^3^/E^3^) where Z is the atomic number and E is the energy of the photons^3,17^. The probability of the photoelectric effect is proportional to the cube of the atomic number and explains the strong attenuation of the X-rays in materials with high atomic numbers such as metallic hardware in comparison to surrounding soft tissues with low and nearly water-equivalent atomic numbers.

The high absorption of the X-rays in metal leads to an insufficient number of photons at the detectors and statistical errors of the projection data. The phenomenon is called photon starvation that causes particularly bright artefacts^18,19^.

Polychromatic x-rays cause beam hardening. As the X-rays travel through an object, photons with low energy are attenuated more than high energy due to the photoelectric effect. The fraction of high-energy photons increases and the energy spectrum gets shifted towards higher energies. This shift is variable in different projection angles around the scanned object that leads to inconsistent data acquisition with subsequent dark streak artefacts near metal structure in the reconstructed CT image. The artefact is more pronounced if x-rays pass through materials with high atomic number such as metal in comparison to materials with lower atomic numbers such as water due to the higher grade of absorption^20,21^.

There are several approaches to overcome metallic artefacts caused by beam hardening and photon starvation. Prefiltering the polychromatic X-rays by an interposed filter (aluminium, copper, tin) between X-ray source and patient hardens the spectrum and narrows the energy range^20,21^. The beam hardening artefacts are mitigated but due to the insufficient correction this technique is used as an adjunct to other methods. VMS imaging with DECT provides images that mimic those to be generated by true monochromatic X-rays. A monochromatic image should contain no beam hardening artefacts because of the absence of the spectral shifts. Nevertheless, artefacts are still present like in our study due to photon starvation, high-order beam hardening artefacts^20,22^ and the calculation process of VMS from polychromatic images. VMS can be computed from projection data by undergoing a nonlinear raw data-based preprocessing that should lead to complete removal of beam hardening artefacts. Alternatively, the computation of the VMS takes place in the image domain using a linear combination of low and high-energy polychromatic images that leads to an approximation to proper virtual monochromatic images. Thus, certain image artefacts of polychromatic images are transferred into the VMS images, resulting in residual artefacts. The latter technique was used in this study and has to be considered in interpreting residual artefacts^23^. In clinical CT scanners an important step before image reconstruction is the water precorrection of the raw data that causes higher order beam hardening artefacts when a mixture of materials such as water, bone and metals is imaged^22^. Projection-based MAR algorithms correct the corrupted projection data in photon starvation by replacing them with interpolated, well estimated data on the base of uncorrupted measurements. The intervention can take place either in the projection domain or the reconstructed image domain. Multiple iterative calculations may be necessary to obtain a veritable streak artefact-reduced image, but the algorithms may introduce new artefacts due to misinterpretation during the metal segmentation and interpolation process, and errors during the estimation of corrupted data^21^. Currently, the following projection-based MAR algorithms are commercially available: (a) single-energy MAR (SEMAR; Toshiba Medical Systems, Otawara, Japan); (b) MAR for orthopedic implants (O-MAR; Philips Healthcare, Best, the Netherlands); (c) iterative MAR (iMAR; Siemens Healthineers, Forchheim, Germany); and (d) smart MAR (Smart MAR; GE Healthcare, Milwaukee, U.S.A.).

### Metal implants

The three metal implants compared had different artefact expansion. At equal energies, stainless steel and chrome-cobalt show more severe artefacts than titanium. Titanium absorbs weaker than stainless steel and chrome-cobalt due to the lower linear attenuation coefficient. The differences in LAC (Fig. 3) are most evident with energy levels between 40 and 100 keV. The amount of X-ray absorption depends on the atomic number of the metal, the photon energy and the mass density of the material (i.e., mass attenuation coefficient). A lower photon energy and higher mass density or atomic number of material lead to an increased LAC with subsequent more pronounced artefacts^24^. As a result, titanium implants with polychromatic X-rays cause more beam hardening than chrome-cobalt and stainless-steel implants that can be corrected by VMS^25^. Between 120 and 190 keV the decline of the LAC curves flattens and the curve progressions parallel the artefact extension of each alloy. Since residual artefacts persist even with the titanium alloy, other reasons than beam hardening must be present such as high-order beam hardening artefacts and synthesis of VMS in the imaging domain.

A similar outcome was observed in the presence of stainless steel and chrome-cobalt alloys. The diagrams of the energy-dependent LAC of both metals (Fig. 3) in comparison to the artefact size measurements (Fig. 4A,B) demonstrate the coherent curves of both properties.

The impact of the diameter of the implant on artefact size is demonstrated by comparing the effects on pellets 2 and 6 towards pellets 3 and 5. The calibre and cross-sectional diameter of the femoral stem are variable due to the cone-shaped geometry with a larger extent more proximally (pellet 2 and 6) than distally (pellets 3 and 5). The thicker the diameter of the stem, the larger is the artefact size.

### DECT/VMS

As pointed out metal artefacts will be reduced by DECT. Metal artefact reduction is based on applying VMS with appropriate energy due to beam hardening whereas photon starvation and scatter artefacts will not be corrected^3,5,6,26^. The reduction of metal artefact is visible for all three implants (titanium, chrome-cobalt and stainless steel) with VMS at higher energies (100–190 keV), which is illustrated in Fig. 4. With higher energies the X-ray beams get harder and this leads to less resorption^3^.

A superior reduction of the metal artefact is seen in the presence of titanium implants. The reason is because titanium causes beam hardening that can be optimally corrected with dual energy CT. The density plot (Figs. 5, 6, 7A) demonstrates that in a range of 100 keV–190 keV the pellets in the artefact area (2,3,5,6) achieve similar CT values as the reference pellets. This means that the residual artefacts do not interfere with the environment anymore. The residual artefacts (Fig. 2C1) are explained by high-order beam hardening, the calculation of VMS on the image domain level and photon starvation. In comparison, artefact correction of the other two metals takes place in the expected manner, but the effect is less pronounced even with higher energies (100 keV–190 keV).

With rising energies, the artefact extent decreases, but also the density of samples due to energy-dependent reduction of the LAC. The density curves of reference pellet 1 (Figs. 5, 6, 7A) and the LAC curve of calcium hydroxyapatite (Fig. 3) demonstrate this phenomenon leading to deteriorated contrast behaviour to the environment. A balance between both issues has to be found and seems to be around 100 keV—120 keV, since no further substantial artefact reduction is to be expected with higher energies.

The comparison between monoenergetic and spectral energies confirms the expected and different outcome with regard to artefact size. A superior imaging quality due to metal artefact reduction was demonstrated with monoenergetic energies (70 keV and 140 keV) than with spectral energies (80 kVp and 140 kVp). The voltage (kVp) is defined as the maximum energy of the photons where the mean energy is considerably lower^6,9^. The effect of beam hardening is explained by the use of polychromatic X rays and minimized with VMS.

### Impact of artefact on environment

The impact of metal artefact on the surrounding of the implant is illustrated in the Fig. 2A–C and is shown in density and density deviation plots of the pellets (Figs. 5, 6, 7) The pellets simulate surrounding tissue with bone-equivalent density. Pellets 1 and 4 always demonstrated similar high densities and small density deviation for all three metal implants since there was no interference with metal artefact. However, the effects of artefacts on pellets 2,3,5,6 were significant, and artefact size correlates to alterations in density and density deviations. Pellets 2 and 6 always faced more severe artefacts than pellets 3 and 5 due the larger diameter of the neighbouring femoral stem. The relationship of artefact size and density or density deviation is coherent and reflected by the effects of changing parameters such as metal composition or X-ray energy level. In correlation to the artefact size behaviour density and density deviation do moderately vary between the energy range of 100 keV–190 keV because most of artefact correction already takes place in the 40 keV to 100 keV range (VMS).

### Differences in DECT

For this study DECT sequential acquisition at low and high kilovolts (80 kVp and 140 kVp) was used. The other types of DECT currently available are rapid kilovoltage switching, Dual-source CT, photon counting detector and multilayer detector and different acquisition protocols are used. A recently presented technique even encompasses DECT clinical application including VMS using data acquired by a single energy CT (SECT) scanner^27,28^. Prior DECT knowledge of differences between low- and high-energy CT is integrated into a deep learning model. A complex neural network incorporates the complex relationship and finally enables the prediction of high-energy data for a given input low-energy CT image. Each technique has its advantages and disadvantages. The sequential acquisition (scan-to-scan) requires the least effort of hardware. The acquisition parameters of both scans have to be adapted to achieve similar noise levels of both scans. The main limitation is the long delay between the two data acquisitions that leads to motion artefacts^9,13,29^.

As pointed out other parameters than VMS imaging influence the grade of artefact reduction such as prefiltering, computation of the VMS on the projection or image domain and application of MAR algorithms. Neither prefiltration of the X-rays nor dedicated MAR algorithms have been applied in our study. The results reflect the effect of VMS imaging on different material compositions of frequently used orthopaedic implants and help radiologists to a basic comprehension of effects in clinical routine.

Comparative studies with varying protocols on different scanners including prefiltration, variation of the energy of the initial polychromatic scans and application of MAR algorithms should be initiated in a future step to achieve a deeper knowledge of the different techniques and their effect on artefact reduction. Thus, a more serious transfer of the ex-vivo results to clinical patient examinations would be possible with regard to the available acquisition and processing techniques entailing potential adjustments in this issue.

## Limitations

Our study concept with phantom measurements has some limitations when adopting it for the clinical reality. The phantoms were probed with fixed parameters by pre-set geometry. The axis of all three implants was parallel to gantry. All metals were implanted centrally in the box and the three implants had the same geometry. We used hip prosthesis, and it is self-evident that with other implants different artefact patterns occur. While motion artefacts do not appear in a fixed phantom, the situation may be different in the human body, particularly using DECT with sequential scan-to-scan technique.

Our phantom models were simple and self-constructed with possible small inaccuracies. However, the limitations are very finite and do not lead to any significant restriction of the result. All three phantoms were comparable to each other proven by similar outcomes in SNR measurements.

Another limitation of this study is the restriction of artefact size mainly to dark and bright artefacts in the surrounding of metal implants. However, both artefacts are considered essential components of artefact characteristics and therefore strongly reflect the major limiting artefacts. Additionally, the disturbances of the environment that are relevant in a clinical setting were included and correlated.

The monochromatic and spectral energies were compared between 70 keV and 80 kVp and between 140 keV and 140 kVp. The intervals between the VMS calculation was defined by the software, thus, an ideal comparison of 80 keV and 80 kVp was not possible.

## Conclusion

This phantom study demonstrates artefact reduction in each of the three different metal implants (titanium, stainless steel and chrome-cobalt) with the help of DECT with VMS and VMS with rising energies. This leads to an increase of image quality and better depiction of surrounding structures. Titanium shows much stronger artefact reduction than stainless steel and chrome-cobalt. The grade of artefact reduction of chrome-cobalt and stainless steel is similar.
